# Supplementary material for: Heat adaptation of phage T7 under an extended genetic code
Source: Virus Evol. 2021 Dec 1;7(2):veab100. doi: 10.1093/ve/veab100 (PMC8923235; doi:10.1093/ve/veab100)
Supplement: veab100_Supp [file veab100_supp.zip › Sequencing_Analysis.zip!/Sequencing_Analysis/A2/evidence/JC_SIDE_2_54.html]

BRESEQ :: Evidence


| New junction evidence | | | | | | | | | | | |
| --- | --- | --- | --- | --- | --- | --- | --- | --- | --- | --- | --- |
|  | | seq id | position | reads (cov) | reads (cov) | score | skew | freq | annotation | gene | product |
| \* | ? | NC\_001604.1 | = 16476 | 362 (0.790) | 39 (0.090) | 31/590 | 11.1 | 9.2% | intergenic (–/–) | *–/–* | –/– |
|  | ? | NC\_001604.1 | 17143 = | 418 (0.930) |  |  |  |  | intergenic (–/–) | *–/–* | –/– |
| Rejected: Coverage evenness skew score above cutoff. | | | | | | | | | | | |
| Rejected: Frequency below/above cutoff threshold. | | | | | | | | | | | |

Only 100 of 435 total aligned reads displayed.  

`ATTATTATGGCTATGACAAAGAAATTTAAAGTGTCCTTCGACGTTACCGCAAAGATGTCGTCTGACGTTCAGGCAATCTTAGAGAAAGATATGCTGCATCTATGTAAGCAGGTCGGCTCAGGTGCGATTGTCCCCAATGGTAAACAGAAGGAAATGATTGTCCAGTTCCTGACACACGGTATGGAAGGATTGATGACATTCGTAGTACGTACATCATTTCGTGAGGCCATTAAGGACATGCACGAAGAGTATGCAGATAAGGACTCTTTCAAACAATCTCCTGCAACAGTACGGGAGGTGTTCTGATGTCTGACTACCTGAAAGTGCTGCAAGCAATCAAAAGTTGCCCTAAGACTTTCCAGTCCAACTATGTACGGAACAATGCGAGCCTCGTAGCGGAGGCCGCTT‑CCCGTGGTCACATCTCGTGCCTGACTACTAGTGGACGTAACGGTGGCGCTTGGGAAATCACTGCTTCCGGTACTCGCTTTCTGAAACGAATGGGAGGATG‑TGTCTAATGTCTCGTGACCTTGTGACTATTCCACGCGATGTGTGGAACGATATACAGGGCTACATCGACTCTCTGGAACGTGAGAACGAT  >  NC_001604.1/16845‑17442`  

                                                                                                                                                                                                                                                                                                          `|`                                                                                                                                                                                                                                                                                                               
   `attatGGCTATGACAAAGAAATTTAAAGTGTCCTTCGACGTTACCGCAAAGATGTCGTCTGACGTTCAGGCAATCTTAGAGAAAGATATGCTGCATCTATGTAAGCAGGTCGGCTCAGGTGCGATTGTCCCCAATGGTAAACAGAAGGAAATGATTGTCCAGTTCCTGACACACGGTATGGAAGGATTGATGACATTCGTAGTACGTACATCATTTCGTGAGGCCATTAAGGACATGCACGAAGAGTATGCAGATAAGGACTCTTTCAAACAATCTCCTGCAACAGTACGGGAGGTGTTcc                                                                                                                                                                                                                                                                                                          >  1:668755/1‑300 (MQ=255)`  
    `ttatGGCTATGACAAAGAAATTTAAAGTGTCCTTCGACGTTACCGCAAAGATGTCGTCTGACGTTCAGGCAATCTTAGAGAAAGATATGCTGCATCTATGTAAGCAGGTCGGCTCAGGTGCGATTGTCCCCAATGGTAAACAGAAGGAAATGATTGTCCAGTTCCTGACACACGGTATGGAAGGATTGATGACATTCGTAGTACGTACATCATTTCGTGAGGCCATTAAGGACATGCACGAAGAGTATGCAGATAAGGACTCTTTCAAACAATCTCCTGCAACAGTACGGGAGGTGTTCTg                                                                                                                                                                                                                                                                                                         <  1:284534/301‑1 (MQ=255)`  
     `cagggCTATGACAAAGAAATTTAAAGTGTCCTTCGACGTTACCGCAAAGATGTCGTCTGACGTTAAGGCAATCTTAGAGAAAGATATGCTGCATCTATGTAAGCAGGTCGGCTCAGGTGCGATTGTCCACAATGGTAAACAGAAGGAAATGATTGTCCAGTTCCTGACACACGGTATGGAAGGATTGATGACATTCGTAGTACGTACATCATTTCGTGAGGCCATTAAGGACATGCACGAAGAGTATGCAGATAAGGACTCTTTCAAACAATCTCCTGCAACAGTACGGGAGGTGTTCTGa                                                                                                                                                                                                                                                                                                        <  1:45810/298‑1 (MQ=255)`  
       `tgacgaTGACAAAGAAATTTAAAGTGTCATTCGACGTTACCGCAAATATGTTGTCTGACGTTCAGGCAATCTTAGAGAAAGATATGCTGCATCTATGTAAGCAGGTCGGCTCAGGTGCGATTGTCCCCAATGGTAAACAGAAGTAAATGATTGTCCAGTTCCTGACACACGGTATGGAAGGATTGATGACATTCGTAGTACGTACATCATTTCGTGAGGCCATTAAGGACATGCACGAAGAGTATGCAGATAAGGACTCTTTCAAACAATCTCCTGCAACAGTACGGGAGGTGTTCTGATg                                                                                                                                                                                                                                                                                                      <  1:380882/296‑1 (MQ=255)`  
        `ggCTATGACAAAGAAATTTAAAGTGTCCTTCGACGTTACTGCAAAGATGTCGTCTGACGTTCAGGCAATCTTAGAGAAAGATATGCTGCATCTATGTAAGCAGGTCGGCTCAGGTGCGATTGTCCCCAATGGTAAACAGAAGGAAATGATTGTCCAGTTCCTGACACACGGTATGGAAGGATTGATGACATTCGTAGTACGTACATCATTTCGTGAGGCCATTAAGGACATGCACGAAGAGTATGCAGATAAGGACTCTTTCAAACAATCTCCTGCAACAGTACGGGAGGTGTTcctgtct                                                                                                                                                                                                                                                                                                     >  1:688951/1‑295 (MQ=255)`  
                       `aagagacaggtCCTTCGACGTTACCGCAAAGATGTCGTCTGACGTTCAGGCAATCTTAGAGAAAGATATGCTGCATCTATGTAAGCAGGTCGGCTCAGGTGCGATTGTCCCCAATGGTAAACAGAAGGAAATGATTGTCCAGTTCCTGACACACGGTATGTAAGGATTGATGACATTCGTAGTATGTACATCATTTCGTGAGGCCATTAAGGACATGCACGAATAGTATGCAGATAAGGACTCTTTCAAACAATCTCCTGCAACAGTACGGGAGGTGTTCTGATGTCTGACTACCTGAAAg                                                                                                                                                                                                                                                                                      <  1:816447/292‑1 (MQ=255)`  
                       `aTTTAAAGTGTCCTTCGACGTTACCGCAAAGATGTCGTCTGACGTTCAGGCAATCTTAGAGAAAGATATGCTGCATCTATGTAAGCAGGTCGGCTCAAGTGCGATTGTCCCCAATGGTAAACAGAAGGAAATGATTGTCCAGTTCCTGACACACGGTATGGAAGGATTGATGACATTCGTAGTACGTACATCATTCCGTGAGGCCATTAAGGACATGCACGAAGAGTATGCAGATAAGGACTCTTTCAAACAATCTCCTGCAACAGTACGGGAGGTGTTCTGATGTCTGACTACCTGAAAg                                                                                                                                                                                                                                                                                      <  1:504742/301‑1 (MQ=255)`  
                                             `aCCGCAAAGATGTCGTCTGACGTTCAGGCAATCTTAGAGAAAGATATGCTGCATCTATGTAAGCAGGTCGGCTCAGGTGCGATTGTCCCCAATGGTAAACAGAAGGAAATGATTGTCCAGTTCCTGACACACGGTATGGAAGGATTGATGACATTCGTAGTACGTACATCATTTCGTGAGGCCATTAAGGACATGCACGAAGAGTATGCAGATAAGGACTCTTTCAAACAATCTACTGCAACAGTACGGGAGGTGTTCTGATATCTGACTACCTgctgtctcttatacacatctccgagcc                                                                                                                                                                                                                                                                >  1:392805/1‑275 (MQ=255)`  
                                              `ccGCAAAGATGTCGTCTGACGTTCAGGCAATCTTAGAGAAAGATATGCTGCATCTATGTAAGCAGGTCGGCTCAGGTGCGATTGTCCCCAATGGTAAACAGAAGGAAATGATTGTCCAGTTCCTGACACACGGTATGGAAGGATTGATGACATTCGTAGTACGTACATCATTTCGTGAGGCCATTAAGGACATGCACGAAGAGTATGCAGATAAGGACTCTTTCAAACAATCTCCTGCAACAGTACGGGAGGTGTTCTGATGTCTGACTACCTGAAAGTGCTGCAAGCAATCAAAAGTTGc                                                                                                                                                                                                                                                               >  1:198157/1‑301 (MQ=255)`  
                                                   `aaGATGTCGTCTGACGTTCAGGCAATCTTAGAGAAAGATATGCTGCATCTATGTAAGCAGGTCGGCTCAGGTGCGATTGTCCCCAATGGTAAACAGAAGGAAATGATTGTCCAGTTCCTGACACACGGTATGGAAGGATTGATGACATTCGTAGTACGTACATCATTTCGTGAGGCCATTAAGGACATGCACGAAGAGTATGCAGATAAGGACTCTTTCAAACCATCTCCTGCAACAGTACGGGAGGTGTTCTGATGTCTGACTACCTGAAAGTGCTGCAAGCAATCAAAAGTTCCCCTaa                                                                                                                                                                                                                                                          >  1:279340/1‑301 (MQ=255)`  
                                                      `aTGTCGTCTGACGTTCAGGCAATTTTAGAGAAAGATATGCTGCATCTATGTAAGCAGGTCGGCTCAGGTGCGATTGTCCCCAATGGTAAACAGAAGGAAATGATTGTCCAGTTCCTGACACACGGTATGGAAGGATTGATGACATTCGTAGTACGTACATCATTTCGTGAGGCCATTAAGGACATGCACGAAGAGTATGCAGATAAGGACTCTTTCAAACAATCTCCTGCAACAGTACGGGAGGTGTTCTGATGTCTGACTACCTGAAAGTGCTGCAAGCAATCAAAAGTTGCCCTAAGAc                                                                                                                                                                                                                                                       <  1:495898/301‑1 (MQ=255)`  
                                                                     `cAGGCAATCTTAGAGAAAGATATGCTGCATCTATGTAAGCAGGTCGGCTCAGGTGCGATTGTCCCCAATGGTAAACAGAAGGAAATGATTGTCCAGTTCCTGACACACGGTATGGAAGGATTGATGACATTCGTAGTACGTACATCATTTCGTGAGGCCATTAAGGACATGCACGAAGAGTATGCAGATAAGGACTCTTTCAAACAATCTACTGCAACAGTACGGGAGGTGTTCTGATGTCTGACTACCTGAAAGTGCTGCAAGCAATCAAAAGTTGCCCTAAGACTTTCCAGTCCAACTa                                                                                                                                                                                                                                        >  1:361895/1‑301 (MQ=255)`  
                                                                     `ataagagacagagagAAAGATATGCTGCATCTATGTAAGCAGGTCGGCTCAGGTGCGATTGTCCACAATGGTAAACAGAAGGAAATGATTGTCCAGTTCCTGACACACGGTATGGAAGGATTGATGACATTCGTAGTACGTACATCATTTCGTGAGGCCATTAAGGACATGCACGAAGAGTATGCAGATAAGGACTCTTTCAAACAATCTCCTGCAACAGTACGGGAGGTGTTCTGATGTCTGACTACCTGAAAGTGCTGCAAGCAATCAAAAGTTGCCCTAAGACTTTCCAGTCCAACTa                                                                                                                                                                                                                                        <  1:715399/290‑1 (MQ=255)`  
                                                                       `ggCAATCTTAGAGAAAGATATGCTGCATCTATGTAAGCAGGTCGGCTCAGGTGCGATTGTCCCCAATGGTAAACAGAAGGAAATGATTGTCCAGTTCCTGACACACTGTATGGAAGGATTGATGACATTCGTAGTACGTACATCATTTCGTGAGGCCATTAATGACATGCACGAAGAGTATGCAGATAAGGACTCTTTCAAACAATCTCCTGCAACAGTACGGGAGGTGTTCTGATGTCTGACTACCTGAAAGTGCTGCAAGCAATCAAAAGTTGCCCTAAGACTTTCCAGTCCAACTAtt                                                                                                                                                                                                                                      >  1:803905/1‑300 (MQ=255)`  
                                                                          `aaTCTTAGAGAAAGATATGCTGCAGCTATGTAAGCAGGTCGGCTCAGGTGCGAGTGTCCCCAATGGTAAACAGAAGGAAATGATTGTCCAGTTCCTGACACACGGTATGGAAGGATTGATGACATTCGTAGTACGTACATCATTTCGTGAGGCCATTAAGGACATGCACGAAGAGTATGCAGATAAGGACTCTTTCAAACAATCTCCTGCAACAGTACGGGAGGTGTTCTGATGTCTGACTACCTGAAAGTGCTGCAAGCAATCAAAAGTTGCCCTAAGACTTTCCAGTCCAACTATGTAc                                                                                                                                                                                                                                   <  1:556876/301‑1 (MQ=255)`  
                                                                           `aTCTTATAGAAAGATATGCTGCATCTATGTAAGCAGGTCGGCTCAGGTGCGATTGTCCCCAATGGTAAACAGAAGGAAATGATTGTCCAGTTCCTGACACACGGTATGGAAGGATTGATGACATTCGTAGTACGTACATCATTTCGTGAGGCCATTAAGGACATGCACGAAGAGTATGCAGATAAGGACTCTTTCAAACAATCTCCTGCAACAGTACGGGAGGTGTTCTGATGTCTGACTACCTGAAAGTGCTGCAAGCAATCAAAAGTTGCCCTAAGACTTTCCAGTCCAACTATGTACg                                                                                                                                                                                                                                  >  1:61518/1‑301 (MQ=255)`  
                                                                           `aTCTTAGAGAAAGATATGCTGCATCTATGTAAGCAGGTCGGCTCAGGTGCGATTGTCCCCAATGGTAAACAGAAGGAAATGATTGTCCAGTTCCTGACACACGGTATGGAAGGATTGATGACATTAGTAGTACGTACATCATTTCGTGAGGCCATTAAGGACATGCACAAAGAGTATGCAGATAAGGACTCTTTCAAACAATCTCCTGCAACAGTACGGGAGGTGTTCTGATGTCTGACTACCTGAAAGTGCTGCAAGCAATCAAAAGTTGCCCTAAGACTTTCCAGTCCAACTATGTACg                                                                                                                                                                                                                                  >  1:756854/1‑301 (MQ=255)`  
                                                                               `aagagAAAGATATGCTGCATCTATGTAAGCAGGTCGGCTCAGGTGCGAATGTCCCCAATGGTAAACAGAAGGAAATGATTGTCCAGTTCCTGACACACTGTATGGAAGGATTGATGACATTCGTAGTACGTACATCAGTTCGTGAGGCCATTAAGGACATGCACGAAGAGTATGCAGATAAGGACTCTTTCAAACAATCTCCTGCAACAGTACGGGAGGTGTTCTGATGTCTGACTACCTGAAAGTGCTGCAAGCAATCAAAAGTTGCCCTAAGACTTTCCAGTCCAACTATGTACGGAAc                                                                                                                                                                                                                              <  1:533638/300‑1 (MQ=255)`  
                                                                                   `aaaaGATATGCTGCATCTATGTAAGCAGGTCGGCTCAGGTGCGATTGTCCCCAATGGTAAACAGAAGGAAATGATTGTCCAGTTCCTGACACACGGTATGGAAGGATTGATGACATTCGTAGTACGTACATCATTTCGTGAGGCCATTAAGGACATGCACGAATAGTATGTAGATAAGGACTCTTTCAAACAATCTCCTGCAACAGTACGGGAGGTGTTCTGATGTCTGACTACCTGAAAGTGCTGCAAGCAATCAAAAGTTGCCCTAAGACTTTCCAGTCCAACTATGTACGGAACAATg                                                                                                                                                                                                                          <  1:597270/300‑1 (MQ=255)`  
                                                                                    `acaGATATGCTGCATCTATGTAAGCAGGTCGGCTCAGGTGCGATTGTCCCCAATGGTAAACAGAAGGAAATGATTGTCCAGTTCCTGACACACGGTATGGAAGGATTGATGACATTCGTAGTACGTACATCATTTCGTGAGGCCATTAAGGACATGCACGAAGAGTATGCAGATAAGGACTCTTTCAAACAATCTCCTGCAACAGTACGGGAGGTGTTCTGATGTCTGACTACCTGAAAGTGCTGCAAGCAATCAAAAGTTGCCCTAAGACTTTCCAGTCCAACTATGTACGGAACAATGc                                                                                                                                                                                                                         <  1:682547/299‑1 (MQ=255)`  
                                                                                       `gATATGCTGCATCTATGTAAGCAGGTCGGCTCAGGTGCGATTGTCCCCAATGGTAAACAGAAGGAAATGATTGTCCAGTTCCTGACACACGGTATGGAAGGATTGATGACATTCGTAGTACGTACATCATTTCGTGAGGCCATTAAGGACATGCACGAAGAGTATGCAGATAATGACTCTTTCAAACAATCTCCTGCAACAGTACGGGAGGTGTTCTGATGTCTGACTACCTGAAAGTGCTGCAAGCAATCAAAAGTTGCCCTAAGACTTTCCAGTCCAACTATGTACGGactgtctcttt                                                                                                                                                                                                                      >  1:152490/1‑291 (MQ=255)`  
                                                                                           `tgctgcATCTATGTAAGCAGGTCGGCTCAGGTGCGATTGTCCCCAATTGTAAACAGAAGGAAATGATTGTCCAGTTCCTGACACACGGTATGGAAGGATTGATGACATTCGTAGTACGTACATCATTTCGTGAGGCCATTAAGGACATGCACGAAGAGTATGCAGATAAGGACTCTTTCAAACAATCTCCTGCAACAGTACGGGAGGTGTTCTGATGTCTGACTACCTGAAAGTGCTGCAAGCAATCAAAAGTTGCCCTAAGACTTTCCAGTCCAACTATGTACGGAACAATGCGAGCCTc                                                                                                                                                                                                                  >  1:819537/1‑301 (MQ=255)`  
                                                                                                `tgtataagagacagggTCGGCTCAGGTGCGATTGTCCCCAATGGTAAACAGAAGGAAATGATTGTCCAGTTCCTGACACACGGTATGGAAGGATTGATGACATTCGTAGTACGTACATCATTTCGTGAGGCCATTAAGGACATGCACGAAGAGTATGCAGATAAGGACTCTTTCAAACAATCTCCTGCAACAGTACGGGAGGTGTTCTGATGTCTGACTACCTGAAAGTGCTGCAAGCAATCAAAAGTTGACCTAAGACTTTCCAGTCCAACTATGTACGGAACAATGCGAGCCTCGTAGc                                                                                                                                                                                                             <  1:695916/287‑1 (MQ=255)`  
                                                                                                  `tCTATGTAAGCAGGTCGGCTAAGGTGCGATTGTCCCCGATGGTAAACAGAAGGAAATGATTGTCCAGTTCCTGACACACGGTATGGAAGGATTGATGACATTGGTAGTACGTACATCATTTCGTGAGGCCATTAAGGACATGCACGAAGAGTATGCAGATAAGGACTCTTTCAAACAATCTCCTGCAACAGTACGGGAGGTGTTCTGATGTCTGACTACCTGAAAGTGCTGCAAGCAATCAAAAGTTGCCCTAAGACTTTCCAGTCCAACTATGTACGGAACAATGCAAGCCTCGTAGCgg                                                                                                                                                                                                           <  1:167833/301‑1 (MQ=255)`  
                                                                                                  `gagatgtgtataagagacagcAGTTGCGATTGTCCCCAATGGTAAACAGAAGGAAATGATTGTCCAGTTCCTGACACGCGGTATGGAAGGATTGATGACATTCGTAGTACGTACATCATTTCGTGAGGCCATTAAGGACATGCACGAAGAGTATGCAGATAAGGACTCTTTCAAACAATCTCCTGCAACAGTACGGGAGGTGTTCTGATGTCTGACTACCTGAAAGTGCTGCAAGCAATCAAAAGTTGCCCTAAGACTTTCCAGTCCAACTATGTACTGAACAATTCGAGCCTCGTAGCgg                                                                                                                                                                                                           <  1:744390/281‑1 (MQ=255)`  
                                                                                                     `aTGTAAGCAGGTCGGCTCATGTGCGATTGTCCCCAATGGTAAACAGAAGGAAATGATTGTCCAGTTCCTGACACACGGTATGGAAGGATTGATGACATTCGTAGTACGTACATCATTTCGTGAGGCCATTAAGGACATGCACGAAGAGTATGCAGATAAGGACTCTTTCAAACAATCTCCTGCAACAGTACTGGAGGTGTTCTGATGTCTGACTACCTGAAAGTGCTGCAAGCAATCAAAAGTTGCCCTAAGACTTTCCAGTCCAACTATgctgtctcttatacacatctccgagcccacc                                                                                                                                                                                                        >  1:794661/1‑271 (MQ=255)`  
                                                                                                     `aTGTAAGCAGGTCGGCTCAGGTGCGATTGTCCCCAATGGTAAACAGAAGGAAATGATTGTCCAGTTCCTGACACACGGTATGGAAGGATTGATGACATTCGTAGTACGTACATCATTTCGTGAGGCCATTAAGGACATGCACGAAGAGTATGCAGATAAGGACTCTTTCAAACAATCTCCTGCAACAGTACGGGAGGTGTTCTGATGTCTGACTACCTGAAAGTGCTTCAAGCAATCAAAAGTTGCCCTAAGACTTTCCAGTCCAACTATGTACGGAACAATGCGAGCCTTGTAGCGGAgg                                                                                                                                                                                                        >  1:616946/1‑301 (MQ=255)`  
                                                                                                      `tGTAAGCAGGTCGTCTCAGGTGCGATTGTCCCCAATGGTAAACAGAAGGAAATGATTGTCCAGTTCCTGACACACGGTATGGAAGGATTGATGACATTCGTAGTACGTACATCATTTCGTGAGGCCATTAAGGACATGCACGAAGAGTATGCAGATAAGGACTCTTTCAAACAATCTCCTGCAACAGTACGGGAGGTGTTCTGATGTCTGACTACCTGAAAGTGCTGCAAGCAATCAAAAGTTGCCCTAAGACTTTCCAGTCCAACTATGTACGGAACAATGCGAGCCTCGTAGCGGAGGc                                                                                                                                                                                                       <  1:278447/301‑1 (MQ=255)`  
                                                                                                                          `gggctcggagatgtgtataagagacagggAAATGATTGTCCAGTTCCTGACACACGGTATGGAAGGATTGATGACATTCGTAGTACGTACATCATTTCGTGAGGCCATTAAGGACATGCGCGAAGAGTATGCAGATAAGGACTCTTTCAAACAATCTCCTGCAATAGTACGGGAGGTGTTCTGATGTCTGACTACCTGAAAGTGCTGCAAGCAATCAAAAGTTGCCCTAAGACTTTCCAGTCCAACTATGTACGGAACAATGCGAGCCTCGTAGCGGAGGCCGCTT‑CCCGTGGTCACAtct                                                                                                                                                                                  <  1:539126/274‑1 (MQ=255)`  
                                                                                                                          `ggCGATTGTCCCCAATGGTAAACAGAAGGAAATGATTGTCCAGTTCCTGACACACGGTATGGAAGGATTGATGACATTCGTAGTACGTACATCATTTCGTGAGGCCATTAAGGACATGCACGAAGAGTATGCAGATAAGGACTCTTTCAAACAATCTCCTGCAACAGTACGGGAGGTGTTCTGATGTCTGACTACCTGAAAGTGCTGCAAGCAATCAAAAGTTGCCCTAAGACTTTCAAGTCCAACTATGTACGGAACAATGCGAGCCTCGTAGCGGAGGCCGCTT‑CCCGTGGTCACAtct                                                                                                                                                                                  <  1:236460/300‑1 (MQ=255)`  
                                                                                                                              `aTTGTCCCCAATGGTAAACAGAAGGAAATGATTGTCCAGTTCCTGACACACGGTATGGAAGGATTGATGACATTCGTAGTACGTACATCATTTCGTGAGGCCATTAAGGACATGCACGAAGAGTATGCAGATAAGGACTCTTTCAAACAATCTCCTGCAACAGTACGGGATGTGTTCTGATGTCTGACTACCTGAAAGTGCTGCAAGCAATCAAAAGTTGCCCTAAGACTTTCCAGTCCAACTATGTACGGAACAATGCGAGCCTCGTAGCGGAGGCCGCTT‑CCCATGGTCACATCTCGTg                                                                                                                                                                              <  1:766351/301‑1 (MQ=255)`  
                                                                                                                               `agatgtttataagagacaggAAGTAAATGGTTTTCCAGTTCCTGACACACGGTATGGAAGGATTGATGACATTCGTAGTACGTACATCATTTCGTGAGGCCATTAAGGACATGCACGAAGATTATGCAGATAAGGACTCTTTCAAACAATCTCCTGCAACAGTACGGGAGGTGTTCTGATGTCTGACTACCTGAAAGTGCTGCAAGCAATCAAAAGTTGCCCTAAGTCTTTCAAGTCCAACTATGTACGGAACAATGCGAGCCTCGTAGCGGAGGCCGCTT‑CCCGTGGTCACATCTCGTGc                                                                                                                                                                             <  1:213428/282‑1 (MQ=255)`  
                                                                                                                                   `ccccAATGGTAAACAGAAGGAAATGATTGTCCAGTTCCTGACACACGGTATGGAAGGATTGATGACATTCGTAGTACGTACATCATTTCGTGAGGCCATTAAGGACATTCACGAAGAGTATGCAGATAATGACTCTTTCAAACAATCTCCTGCAACAGTACGGGAGGTGTTCTGATGTCTGACTACCTGAAAGTGCTGCAAGCAATCAAAAGTTGCCCTAAGACTTTCCAGTCCAACTATGTACGGAACAATGCGAGCCTCGTAGCGGAGGCCGCTT‑CCCGTGGTCACATCTCGTGCCTGa                                                                                                                                                                         >  1:653306/1‑301 (MQ=255)`  
                                                                                                                                       `aaTGGTAAACAGAAGGAAATGATTGTCCAGTTCCTGACACACGGTATGGAAGGATTGATGACATTCGTAGTACGTACATCATTTCGTGAGGCCATTAAGGACATGCACGAAGAGTATGCAGATAAGGACTCTTTCAAACAATCTCCTGCAACAGTACGGGAGGTGTTCTGATGTCTGACTACCTGAAAGTGCTGCAAGCAATCAAAAGTTGCCCTAAGACTTTCCAGTCCAACTATGTACGGAACAATGCGAGCCTCGTAGCGGAGGCCGCTT‑CCCGTGGTCACATCTCGTGCCTGactac                                                                                                                                                                     <  1:31306/301‑1 (MQ=255)`  
                                                                                                                                        `aTGGTAAACATAAGGAAATGATTGTCAAGTTCCTGACACACGGTATGGAAGGATTGATGACATTCGTAGTACGTACATCATTTCGTGAGGCCATTAAGGACATGCACGAAGAGTATGCAGATAAGGACTCTTTCAAACAATCTCCTGCAACAGTACGGGAGGTGTTCTGATGTCTGACTACCTGAAAGTGCTGCAAGCAATCAAAAGTTGCCCTAAGACTTTCCAGTCCAACTATGTACGGAACAATGCGAGCCTCGTAGCGGAGGCCGCTT‑CCCGTGGTCACATCTCGTGCCTGactact                                                                                                                                                                    >  1:591878/1‑301 (MQ=255)`  
                                                                                                                                            `tAAACAGAAGGAAATGATTGTCCAGTTCCTGACACACGGTATGGAAGGATTGATGACATTCGTAGTACGTACATCATTTCGTGAGGCCATTAAGGACATGCACGAAGAGTATGCAGATAATGACTCTTTCAAACAATCTCCTGCAACAGTACGGGAGGTGTTCTGATGTCTGACTACCTGAAAGTGCTGCAAGCAATCAAAAGTTGCCCTAAGACTTTCCAGTCCAACTATGTACGGAACAATGCGAGCCTCGTAGCGGAGGCCGCTT‑CCCGTGGTCACATCTAGTGCCTGACTACTAGTg                                                                                                                                                                <  1:478761/301‑1 (MQ=255)`  
                                                                                                                                            `tAAACAGAAGGAAATGATTGTCAAGTTCCTGACACACGGTATGGAAGGATTGATGACATTCGTAGTACGTACATCATTTCGTGAGGCCATTAAGGACATGCACGAAGAGTATGCAGATAAGGACTCTTTCAAACAATCTCCTGCAACAGTACGGGAGGTGTTCTGATGTCTGACTACCTGAAAGTGCTGCAAGCAATCAAAAGTTGCCCTAAGACTTTCCAGTCCAACTATGTACGGAACAATGCAAGCCTCGTAGCGGAGGCCGCTT‑CCCGTGGTCACATCTCGTGCCTGACTACTAGTg                                                                                                                                                                <  1:374849/301‑1 (MQ=255)`  
                                                                                                                                             `aaaCAGAAGGAAATGATTGTCCAGTTCCTGACACACGGTATGGAAGGATTGATGACATTCGTAGTACGTACATCATTTCGTGAGGCCATTAAGGACATGCACGAAGAGTATGCAGATAAGGACTCTTTCAAACAATCTCCTGCAACAGTACTGGAGGTGTTCTGATGTCTGACTACCTGAAAGTGCTGCAAGCAATCAAAAGTTGCCCTAATACTTTCCAGTCCAACTATGTACGGAACAATGCGAGCCTCGTAGCGGAGGCCGCTT‑CCCGTGGTCACATCTCGTGCCTGACTACTAGTgg                                                                                                                                                               >  1:102111/1‑301 (MQ=255)`  
                                                                                                                                                      `gctgactcagagtaaagaggacttggttgTATTGAAGGATTGATGAAATTCGTAGTAAGTACATCATTTCGTGAGGCCATTAAGGACATGCACGAAGAGTATGCAGATAAGGACTCTTTCAAACAATCTCCTGCAACAGTACGGTAGGTGTTCTGATGTCTGACTACCTGAAAGTGCTGCAAGCAATCAAAAGTTGCCCTAAGACTTTCCAGGCCAACTATGTACGGAACAATGCGAGCCTCGTATCGGAGGCCGCTT‑CCCGTTGTCACATCTCGTGCCTGACTACTAGTGGACGTAACgg                                                                                                                                                      <  1:343393/273‑1 (MQ=255)`  
                                                                                                                                                            `gggagatgtgtataagagacaggTATTGAAGGATTGATTACATTCGTAGTACGTACATCATTTCGTGAGGCCATTAAGGACATGCACGAAGAGTATGCAGATAAGGACTATTTCAAACAATCTACTGCAACAGTACGGGAGGTGTTCTGATGTCTGACTACCTGAAAGTGCTGCAAGCAATCAAAAGTTGCCCTAAGACTTTCCAGTCCAACTATGTACGGAACAATGCGAGCCTCGTAGCGGAGGCCGCTT‑CCCGTGGTCACATCTCGTGCCTGACTACTAGTGGACGTAACGGTGgcgc                                                                                                                                                <  1:611344/280‑1 (MQ=255)`  
                                                                                                                                                            `cggagatgtgtataagagacaggTATGGAAGGATTGATGACATTCGTAGTACGTACATCATTTCGTGAGGCCATTAAGGACATGCACGAAGAGTATGCAGATAAGGACTCTTTCAAACAATCTCCTGCAACAGTACGGGAGGTGTTCTGATGTCTGACTACCTGAAAGTGCTGCAAGCAATCAAAAGTTTCCCTAAGACTTTCCAGTCCAACTATGTACGGAACAATGCGAGCCTCGTAGCGGAGGCCGCTT‑CCCGTGGTCACATCTCGTGCCTGACTACTAGTGGACGTAACGGTGgcgc                                                                                                                                                <  1:451498/280‑1 (MQ=255)`  
                                                                                                                                                                      `tCCTGACACACGGTATGGAAGGATTGATGACATTCGTAGTACGTACATCATTTCGTGAGGCCATTAAGGACATGCACGAAGAGTATGCAGATAAGGACTCTTTCAAACAATCTCCTGCAACAGTACGGGATGTGTTCTGATGTCTGACTACCTGAAAGTGCTGCAAGCAATCAAAAGTTGCCCTAAGACTTTCCAGTCCAACTATGTACGGAACAATGCGAGCCTCGTAGCGGAGGCCGCTT‑CCCGTGGTCACATCTCGTGCCTGACTACTAgctgtctcttatacacatctccgagcccc                                                                                                                                      >  1:29448/1‑273 (MQ=255)`  
                                                                                                                                                                       `ccTGACACACGGTATGGAAGGATTGATGACATTCGTAGTACGTACATCATTTCGTGAGGCCATTAAGGACATGCACGAAGAGTATGCAGATAAGGACTCTTTCAAACAATCTCCTGCAACAGTACGGGAGGTGTTCTGATGTCTGACTACCTGAAAGTGCTGCAAGCAATCAAAAGTTGCCCTAAGACTTTCCAGTCCAACTATGTACGGAACAATGCGAGCCTCGTAGCGGAGGCCGCTT‑CCCGTTGTCACATCTCGTGCCTGACTACTAGTGGACGTAACGGTGGCGCTTGGGAAATCa                                                                                                                                     >  1:658879/1‑301 (MQ=255)`  
                                                                                                                                                                          `gACACACGGTATGGAAGGATTGATGACATTCGTAGTACGTACATCATTTCGTGAGGCCATTAAGGACATGCACGAAGAGTATGCAGATAAGGACTCTTTCAAACAATCTCCTGCAACAGTACGGGAGGTGTTCTGATGTCTGACTACCTGAAAGTGCTGCAAGCAATCAAAAGTTGCCCTAAGACTTTCCAGTCCAACTATGTACGGAACAATGCGAGCCTCGTAGCGGAGGCCGCTT‑CACGTGGTCACATCTCGTGCCTGACTACTAGTGGACGTAACGGTGGCGCTTGGGAAATCACTg                                                                                                                                  >  1:579383/1‑301 (MQ=255)`  
                                                                                                                                                                          `gACACACGGTAGGGAAGGATTGATGACATTCGTAGGACGTACATCATTTCGTGAGTCCATGAAGGACATGCACGAAGAGTATGCAGATAAGGACTCTTTCAAACAATCTCCTGCAACAGTACGGGAGGTGTTCTGATGTCTGACTACCTGAAAGTGCTGCAAGCAATCAAAAGTTGCCCTAAGACTTTCCAGTCCAACTATGTACGGAACAATGCGAGCCTCGTAGCGGAGGCCGCTT‑CCCGTGGTCACATCTCGTGCCTGACTACTAGTGGACGTAACGGTGGCGCTTGGGAAATCACTg                                                                                                                                  <  1:398371/301‑1 (MQ=255)`  
                                                                                                                                                                                  `gTATGGAAGGATTGATGACATTCGTAGTACGTACATCATTTCGTGAGGCCATTAAGGACATGCACGAAGAGTATGCAGATAATGACTCTTTCAAACAATCTCCTGCAACAGTACTGGAGGTGTTCTGATGTCTGACTACCTGAAAGTGCTGCAAGCAATCAAAAGTTGCCCTAAGACTTTCCAGTCCAACTATGTACGGAACAATGCGAGCCTCGTAGCGGAGGCCGCTT‑CCCGTGGTCACATCTCGTGCCTGACTACTAGTGGACGTAACGGTGGCGCTTGGGAAATCACTGCTTCCGGt                                                                                                                          >  1:724052/1‑301 (MQ=255)`  
                                                                                                                                                                                  `gTATGGAAGGATTGATGACATTCGTAGTACGTACATCATTTCGTGAGGCCATTAAGGACATGCACGAAGAGTATGCAGATAAGGACTCTTTCAAACAATCTCCTGCAACAGTACGGGAGGTGTTCTGATGTCTGACTACCTGAAAGTGCTGCAAGCAATCAAAAGTTGCCCTAAGACTTTCCAGTCCAACTATGTACGGAACAATGCGAGCCTCGTAGCGGAGGCCGCTT‑CCCGTGGTCACATCTCGTGCCTGACTACTAGTGGACGTAACGGTGGCGCTTGGGAAATCACTGCTTCCggg                                                                                                                          >  1:741648/1‑300 (MQ=255)`  
                                                                                                                                                                                  `gTATGGAAGGATTGATGACATTCGTAGTACGTACATCATTTCGTGAGGCCATTAAGGACATGCACGAAGAGTATGCAGATAAGGACTCTTTCAAACAATCTCCTGCAACAGTACGGGAGGTGTTCTGATGTCTGACTACCTGAAAGTGCTGCAAGCAATCAAAAGTTGCCCTAAGACTTTCCAGTCCAACTATGTACGGAACAATGCGAGCCTCGTAGCGGAGGCCGCTT‑CCCGTGGTCACATCTCGTGCCTGACTACTAGTGGACGTAACGGTGGCGCTTGGGAAATCACTGCTTCCGGt                                                                                                                          >  1:567931/1‑301 (MQ=255)`  
                                                                                                                                                                                           `gATTGATGATATTCGTAGTACGTACATCATTTCGTGAGGCCATTAAGGACATGCACGAAGAGTATGCAGATAAGGACTCTTTCAAACAATCTCCTGCAACAGTACGGGAGGTGTTCTGATGTCTGACTACCTGAAAGTGCTGCAAGCAATCAAAAGTTGCCCTAAGACTTTCCAGTCCAACTATGTACGGAACAATGCGAGCCTCGTAGCGGAGGCCGCTTCCCCGTGGTCACATCTCGTGCCTGACTACTAGTGGACGTAACGGTGGCGCTTGGGAAATCACTGCTTCCGGTACTCGCtt                                                                                                                  >  1:368151/1‑301 (MQ=255)`  
                                                                                                                                                                                           `gATTGATGACATTCGTAGTACGTACATCATTTCGTGAGGCCATTAAGGACATGCACGAAGAGTATGCAGATAAGGACTCTTTCAAACAATCTCCTGCAACAGTACGGGAGGTGTTCTGATGTCTGACTACCTGAAAGTGCTGCAAGCAATCAAAAGTTGCCCTAAGACTTTCCAGTCCAACTATGTACGGAACAATGCGAGCCTCGTAGCGGAGGCCGCTT‑CCCGTGGTCACATCTCGTGCCTGACTACTAGTGGACGTAACGGTGGCGCTTgggctgtctcttatacacatctccgagcc                                                                                                                 >  1:317849/1‑275 (MQ=255)`  
                                                                                                                                                                                               `gatgaCATTCGTAGTACGTACATCATTTCGTGAGGCCATTAAGGACATGCACGAATAGTATGCAGATAAGGACTCTTTCAAACAATCTCCTGCAACAGTACGGTAGGTGTTCTGATGTCTGACTACCTGAAAGTGCTGCAAGCAATCAAAAGTTGCCCTAAGACTTTCCAGTCCAACTATGTACGGAACAATGCGAGCCTCGTAGCGGAGGCTGCTT‑CCCGTGGTCACATCTCGTGCCTGACTACTAGTGGACGTAACGGTGGCGCTTGGGAAATCACTGCTTCCTGtctcttatacacat                                                                                                             >  1:8086/1‑288 (MQ=255)`  
                                                                                                                                                                                                    `cATTCGTAGTACGTACATCATTTCGTGAGGCCATTAAGGACATGCACGAAGAGTATGCAGATAAGGACTCTTTCAAACAATCTCCTGCAACAGTACGTGAGGTGTTCTGATGTCTGACTACCTGAAAGTGCTGCAAGCAATCAAAAGTTGCCCTAAGACTTTCCAGTCCAACTATGTACGGAACAATGCGAGCCTCGTAGCGGAGGCCGCTT‑CCCGTGGTCACATCTCGTGCCTGACTACTAGTGGACGTAACGGTGGCGCTTGGGAAATCACTGCTTCCGGTACTCGCTTTCTGAAACGa                                                                                                        >  1:624113/1‑301 (MQ=255)`  
                                                                                                                                                                                                    `cATTCGTAGTACGTACATCATTTCGTGAGGCCATTAAGGACATGCACGAAGAGTATGCAGATAAGGACTCTTTCAAACAATCTCCTGCAACAGTACGGGAGGTGTTCTGATGTCTTACTACCTGAAAGTGCTGCAAGCAATCAAAAGTTGCCCTAAGACTTTCCAGTCCAACTATGTACGGAACAATGCGAGCCTCGTAGCGGAGGCCGCTT‑CCCGTGGTCACATCTCGTGCCTGACTACTAGTGGACGTAACGGTGGCGCTTGGGAAATCACTGCTTCCGGTACTCGCTTTCTGAAACGa                                                                                                        >  1:431315/1‑301 (MQ=255)`  
                                                                                                                                                                                                            `gtacgtacATCATTTCGTGAGGCCATTAAGGACATGCACAAAGAGTATGCAGATAAGGACTCTTTCAAACAATCTCCTGCAACAGTACGGGAGGTGTTCTGATGTCTGACTACCTGAAAGTGCTGCAAGCAATCAAAAGTTGCCCTAAGACTTTCCAGTCCAACTATGTACGGAACAATGCAAGCCTCGTAGCGGAGGCCGCTT‑CCCGTGGTCACATCTCGTGCCTGACTACTAGTGGACGTAAAGGTGGCGCTTGGGAAATCACTGCTTCCGGTACTCGCTTTCTGAAACGAATGggagg                                                                                                >  1:408735/1‑301 (MQ=255)`  
                                                                                                                                                                                                               `agtacATCATTTCGTGAGGCCATTAAGGACATGCACGAAGAGTATGCAGATAAGGACTCTTTCAAACAATCTCCTGCAACAGTACGGGAGGTGTTCTGATGTCTGACTACCTGAAAGTGCTGCAAGCAATCAAAAGTTGCCATAAGACTTTCCAGTCCAACTATGTACGGAACAATGCGAGCCTCGTAGCGGAGGCAGCTT‑CACGTGGTCACATCTCGTGCCTGACTACTAGTGGACGTAACGGTGGCGCTTGGGAAATCACTGCTTCCGGTACTCGCTTTCTGAAACGAATGGGAGGAtg                                                                                             <  1:525832/300‑1 (MQ=255)`  
                                                                                                                                                                                                                   `aatcatTTCGTGAGGCCATTAAGGACATGCACGAAGAGTATGCAGATAAGGACTCTTTCAAACAATCTCCTGCAACAGTATGGGAGGTGTTCTGATGTCTGACTACCTGAAAGTGCTGCAAGCAATCAAAAGTTGCCCTAAGACTTTCCAGTCCAACTATGTACGTAACAATGCGAGCCTCGTAGCGGAGGCCGCTT‑CCCGTGGTCACATCTCGTGCCTGACTACTAGTGGACGTAACGGTGGCGCTTGGGAAATCACTGCTTCCGGTACTCGCTTTCTGAAACGAATGGGAGGATG‑TGTc                                                                                        <  1:227646/300‑1 (MQ=255)`  
                                                                                                                                                                                                                         `ttCGTGAGGCCATTAAGGACATGCACGAAGAGTATGCAGATAAGGACTCTTTCAAACAATCTCCTGCAACAGTACGGGAGGTGTTCTGATGTCTGACTACCTGAAAGTGCTGCAAGCAATCAAAAGTTGCCATAAGACTTTACAGTCCAACTATGTACGGAACAATGCGAGCCTCGTAGCGGAGGCCGCTT‑CCCGTGGTCACATCTCGTGCCTGACTACTAGTGGACGTAACGGTGGCGCTTGGGAAATAACTGCTTCCGGTACTCGCTTTCTGAAACGAATGGGAGGATG‑TGTCTAATGt                                                                                  <  1:137565/301‑1 (MQ=255)`  
                                                                                                                                                                                                                         `gtgggctcggagatgtgtataagagacaggagTATGCAGATAAGGACTCTTTCAAACAATCTCCTGCAACAGTACGGGAGGTGTTCTGATGTCTGACTACCTGAAAGTGCTGCAAGCAATCAAAAGTTGGCCTAAGACTTTCCAGTCCAACTATGTACGGAACAATGCGAGCCTCGTAGCGGAGGCCGCTT‑CCCGTGGTCACATCTCGTGCCTGACTACTAGTGGACGTAACGGTGGCGCTTGGGAAATCACTGCTTCCGGTACTCGCTTTCTGAAACGAATGGGAGGATG‑TGTCTAATGt                                                                                  <  1:2518/272‑1 (MQ=255)`  
                                                                                                                                                                                                                             `gaaGGCCATTAAGGACATGCACGAAGAGTATGCATATAAGGACTCTTTCAAACAACTTCCTGCAACTGTAAGGGAGGTGTTCTGATGTCTGACTACCTGAAAGTGCTGCAATCAATCAAAAGTTGCCCTAAGACTTTCCAGTCCAACTATGTACGTAACAATGCGAGCCTCGTAGCGGAGGCCGCTT‑CCCGTGGTCACATCTCGTGCCTGACTACTAGTGGACGTAATGGTGGCGCTTGGGAAATCACTGCTTCCGGTACTCGCTTTCTGAAACGAATGGGATGATG‑TGTCTAATGTCTCg                                                                              <  1:211892/299‑1 (MQ=255)`  
                                                                                                                                                                                                                                 `gCCATTAAGGACATGCACGAAGAGTATGCAGATAAGGACTCTTTCAAACAATCTCCTGCAACAGTACGGGAGGTGTTCTGATGTCTGACTACCTGAAAGTGCTGCAAGCAATCAAAAGTTTCCCTAAGACTTTCCAGTCCAACTATGTACGGAACAATGCGAGCCTCGTAGCGGAGGCCGCTT‑CCCGTGGTCACATCTCGTGCCTGACTACTAGAGGACGTAACGGTGGCGCTTGGGAAATCACTGCTTCCGGTACTCGCTTTCTGAAACGAATGGGAGGATG‑TGTCtctgtctcttatac                                                                          >  1:164540/1‑288 (MQ=255)`  
                                                                                                                                                                                                                                  `ccATTAAGGACATGCACGAAGAGTATGCAGATAAGGACTCTTTCAAACAATCTCCTGCAACAGTACGGGAGGTGTTCTGATGTCTGACTACCTGAAAGTGCTGCAAGCAATCAAAAGTTGCCCTAAGACTTTCCAGTCCAACTATGTACGGAACAATGCGAGCCTCGTAGCGGAGGCCGCTT‑CCCGTGGTCACATCTCGTGCCTGACTAATAGTGGACGTAACGGTGGCGCTTGGGAAATCACTGCTTCCGGTACTCGCTTTCTGAAACGAATGGGAGGGTG‑TGTCTAATGtctgtctctt                                                                         >  1:323106/1‑294 (MQ=255)`  
                                                                                                                                                                                                                                     `ttAAGGACATGCACGAAGAGTATGCAGATAAGGACTCTTTCAAACAATCTCCTGCAACAGTACGGGAGGTGTTCTGATGTCTGACTACCTGAAAGTGCTGCAAGCAATCAAAAGTTGCCCTAAGACTTTCCAGTCCAACTATGTACGGAACAATGCGAGCCTCGTAGCGGAGGCCGATT‑CCCGTGGTCACATCTCGTGCCTGACTACTAGTGGACGTAACGGTGGCGCTTGGGAAATCACTGCTTCCGGTACTCGCTTTCTGAAACGAATGGGAGGATG‑TGTCTAATGtctgtctcttata                                                                      >  1:223230/1‑291 (MQ=255)`  
                                                                                                                                                                                                                                         `ggACATGCACGAAGAGTATGCAGATAAGGACTCTTTCAAACAATCTCCTGCAACAGTACGGGAGGTGTTCTGATGTCTGACTACCTGAAAGTGCTGCAAGCAATCAAAAGTTGCCCTAAGACTTTCCAGTCCAACTATGTACGGAACAATGCGAGCCTCGTAGTGGAGGCCGCTT‑CCCGTGGTCACATCTCGTGCCTGACTACTAGTGGACGTAACGGTGGCGCTTGGGAAATCACTGCTTCCGGTACTCGCTTTCTGAAACGAATGGGAGGATG‑TGTCTAATGTCTCGTGACCTTGGGAc                                                                  <  1:183700/301‑1 (MQ=255)`  
                                                                                                                                                                                                                                              `tGCACGAAGAGTATGCAGATAATGACTCTTTCAAACAATCTCCTGCAACAGTACGGGAGGTGTTCTGATGTCTGACTACCTGAAAGTGCTGCAAGCAATCAAAAGTTGCCCTAATACTTTCCAGTCCAACTATGTACGGAACAATGCGAGCCTCGTAGCGGAGGCCGCTT‑CCCGTGGTCACATCTCGTGCCTGACTACTAGTGGACGTAACGGTGGCGCTTGGGAAATCACTGCTTCCGGTACTCGCTTTCTGAAACGAATGGGAGGATGCTGTCtcttatacacatctccgagcccacga                                                              >  1:601929/1‑276 (MQ=255)`  
                                                                                                                                                                                                                                                `cACGAAGAGTATGCAGATAAGGACTCTTTCAAACAATCTCCTGCAACAGTACGGGAGGTGTTCTGATGTCTGACTACCTGAAAGTGCTGCAAGCAATCAAAAGTTGCCCTAAGACTTTCCAGTCCAACTATGTACGGAACAATGCGAGCCTCGTAGCGGAGGCCGCTT‑CCCGTGGTCACATCTCGTGCCTGACTACTAGTGGACGTAACGGTGGCGCTTGGGAAATCACTGCTTCCGGTACTCGCTTTCTGAAACGAATGGGAGGATG‑TGTCTAATGTCTCGTGACCTTGTGAcctgtctc                                                           >  1:143974/1‑294 (MQ=255)`  
                                                                                                                                                                                                                                                     `agagTATGCAGATAAGGACTCTTTCAAACAATCTCCTGCAATAGTACGGGAGGTGTTCTGATGCCTGACTACCTGAAAGTGCTGCAAGCAATCAAAAGTTGCCCTAAGACTTTCCAGTCCAACTATGTACGGAACAATGCGAGCCTCGTAGCGGAGGCCGCTT‑CCCGTGGTCACATCTCGTGCCTGACTACTAGTGGACGTAACGGTGGCGCTTGGGAAATCACTGCTTCCGGTACTCGCTTTCTGAAACGAATGGGAGGATG‑TGTCTAATGTCTCGTGACCTTGTGACTATTCCACGCGa                                                      >  1:639485/1‑301 (MQ=255)`  
                                                                                                                                                                                                                                                      `gagTATGCAGATAAGGACTCTTTCAAACAATCTCCTGCAACAGTACGGGAGGTGTTCTGATGTCTGACTACCTGAAAGTGCTGCAAGCAATCAAAAGTTGCCCTAAGACTTTCCAGTCCAACTATGTACGGAACAATGCGAGCCTCGTAGCGGAGGCCGCTT‑CCCGTGGTCACATCTCGTGCCTGACTACTAGTGGACGTAACGGTGGCGCTTGGGAAATCACTGCTTCCGGTACTCGCTTTCTGAAACGAATGGGAGGATG‑TGTCTAATGTCTCGTGACCTTGTGACTATTCCACGCGAt                                                     >  1:708687/1‑301 (MQ=255)`  
                                                                                                                                                                                                                                                       `agTATGCAGATAAGGACTCTTTCAAACAATCGCCTGCAACAGTACGGTAGGTGTTCTGATGTCTGACTACCTGAAAGTGCTGCAAGCAATCAAAAGTTGCCCTAAGACTTTCCAGTCCAACTATGTACGGAACAATGCGAGCCTCGTAGCGGAGGCCGCTT‑CCCGTGGTCACATCTCGTGCCTGACTACTAGTGGACGTAACGGGGGCGCTTGGGAAATCACTGCTTCCGGTACTCGCTTTCTGAAACGAATGGGAGGATG‑TGTCTAATGTCTCGTGACCTTGTGACTATTCCACGCGAtg                                                    <  1:412795/301‑1 (MQ=255)`  
                                                                                                                                                                                                                                                        `gTATGCAGATAAGGACTCTTTCAAACAATCTCCTGCAACAGTACGGGAGGTGTTCTGATGTCTGACTACCTGAAAGTGCTGCAAGCAATCAAAAGTTGCCCTAAGACTTTCCAGTCCAACTATGTACGGAACAATGCGAGCCTCGTAGCGGAGGCCGCTT‑CCCGTGGTCACATCTCGTGCCTGACTACTAGTGGACGTAACGGTGGCGCTTGGGAAATCACTGCTTCCGGTACTCGCTTTCTGAAACGAATGGGAGGATG‑TGTCTAATGTCTCGTGACCTTGTGACTATTCCACGCGAttt                                                   >  1:421347/1‑299 (MQ=255)`  
                                                                                                                                                                                                                                                            `gCAGATAAGGACTCTTTCAAACAATCTCCTGCAACAGTACGGGAGGTGTTCTGATGTCTGACTACCTGAAAGTGCTGCAAGCAATCAAAAGTTGCCCTAAGACTTTCCAGTCCAACTATGTACGGAACAATGCGAGCCTCGTAGCGGAGGCCGCTT‑CCCGTGGTCACATCTCGTGCCTGACTACTAGTGGACGTAACGGTGGCGCTTGGGAAATCACTGATTCCGGTACTCGCTTTCTGAAACGAATGGGAGGATG‑TGTCTAATGTCTAGTGACCTTGTGACTATTCCACGCGAtgcttgg                                               >  1:203440/1‑296 (MQ=255)`  
                                                                                                                                                                                                                                                               `gacagggACTCTTTCAAACAATCTCCTGCAACAGTACGGGAGGTGTTCTGATGTCTGACTACCTGAAAGTGCTGCAAGCAATCAAAAGTTGCCCTAAGACTTTCCAGTCCAACTATGTACGGAACAATGCGAGCCTCGTAGCGGAGGCCGCTT‑CCCGTGGTCACATCTCGTGCCTGACTACTAGTGGACGTAACGGTGGCGCTTGGGAAATCACTGCTTCCGGTACTCGCTTTCTGAAACGAATGGGAGGATG‑TGTCTAATGTCTCGTGACCTTGTGACTATTCCACGCGATGTGTGGAAc                                            <  1:484736/296‑1 (MQ=255)`  
                                                                                                                                                                                                                                                               `gATAAGGACTCTTTCAAACAATCTCCTGCAACAGTACGGGAGGTGTTCTGATGTCTGACTACCTGAAAGTGCTGCAAGCAATCAAAAGTTGCCCTAAGACTTTCCAGTCCAACTATGTACGGAACAATGCGAGCCTCGTAGCGGAGTCCGCTT‑CCCGTGGTCACATCTCGTGCCTGACTACTAGTGGACGTAACGGTGGCGCTTGGGAAATCACTGCTTCCGGTACTCGCTTTCTGAAACGAATGGGAGGATG‑TGTCTAATGTCTCGTGACCTTGTGACTATTCCACGCGATGTGTGGAAc                                            >  1:186615/1‑301 (MQ=255)`  
                                                                                                                                                                                                                                                               `gATAAGGACTCTTTCAAACAATCTCCTGCAACAGTACGGGAGGTGTTCTGATGTCTGACTACCTGAAAGTGCTGCAAGCAATCAAAAGTTGCCCTAAGACTTTCCAGTCAAACTATGTACGGAACAATGCGAGCCTCGTAGCGGAGGCCGCTT‑CCCGTGGTCACATCTCGTGCCTGACTACTAGTGGACGTAACGGTGGCGCTTGGGAAATCACTGCTTCCGGTACTCGCTTTCTGAAACGCATGGGAGGATG‑TGTCTAATGTCTCGTGACCTTGTGACTATTCCACGCGATGTGTGGAAc                                            >  1:732447/1‑301 (MQ=255)`  
                                                                                                                                                                                                                                                               `gATAAGGACTCTTTCAAACAATCTCCTGCAACAGTACGGGAGGTGTTCTGATGTCTGACTACCTGAAAGTGCTGCAAGCAATCAAAAGTTGCACTAAGACTTTCCAGTCCAACTATGTACGGAACAATGCAAGCCTCGTAGCGGAGGCCGCTT‑CCCGTGGTCACATCTCGTGCCTGACTACTAGTGGACGTAACGGTGGCGCTTGGGAAATCACTGCTTCCGGTACTCGCTTTCTGAAACGAATGGGAGGATG‑TGTCTAATGTCTCGTGACCTTGTGACTATTCCACGCGATGTGTGGAAc                                            >  1:510475/1‑301 (MQ=255)`  
                                                                                                                                                                                                                                                               `ctcggagatgtgtataagagacagccTGCAACAGTACGGGAGGTGTTATGATGTCTGACTACCTGAAAGTGCTGCAAGCAATCAAAAGTTGCCCTAAGACTTTCCAGTCCAACTATGTACGGAACAATGCGAGCCTCGTAGCGGAGGACGCTT‑CCCGTGGTCACATCTCGTGCCTGACTACTAGTGGACGTAACGGTGGCGCTTGGGAAATCACTGCTTCCGGTACTCGCTTTCTGAAACGAATGGGAGGATG‑TGTCTAATGTCTCGTGACCTTGTGACTATTCCACGCGATGTGTGGAAc                                            <  1:209691/277‑1 (MQ=255)`  
                                                                                                                                                                                                                                                                 `aaaGGACTCTTTCAAACAATCTCCTGCAACAGTACGGGAGGTGTTCTGATGTCTGACTACCTGAAAGTGCTGCAAGCAATCAAAAGTTGCCCTAAGACTTTCCAGTCCAACTATGTACGGAACAATGCGAGCCTCGTAGCGGAGGCCGCTT‑CCCGTGGTCACATCTCATGCCTGACTACTAGTGGACGTAACGGTGGCGCTTGGGAAATCACTGCTTCCGGTACTCGCTTTCTGAAACGAATGGGAGGATG‑TGTCTAATGTCTCGTGACCTTGTGACTATTCCACGCGATGTGTGGAACGa                                          <  1:116218/300‑1 (MQ=255)`  
                                                                                                                                                                                                                                                                   `gtgggctcggagatgtgtataagagacaggTACTGGATGTGTTCTGATGTCTGACTACCTGAAAGTGCTGCAAGCAATCAAAAGTTGCCCTAAGACTTTCCAGTCCAACTATGTACGGAACAATGCGAGCCTCGTAGCGGAGGCCGCTT‑CCCGTGGTCACATCTCGTTCCTGACTACTAGTGGACGTAACGGTGGCGCTTGGGAAATCACTGCTTCCGGTACTCGCTTTCTGAAACGAATGGGAGGATG‑TGTCTAATGTCTCGTGACCTTGTGACTATTCCACGCGATGTGTGGAACGata                                        <  1:238935/272‑1 (MQ=255)`  
                                                                                                                                                                                                                                                                    `tgACTCTTTCAAACAATCTCCTGCAACAGTACGGGAGGTGTTCTGATGTCTGACTACCTGAAAGTGCTGCAAGCAATCAAAAGTTGCCCTAAGACTTTCCAGTCCAACTATGTACGGAACAATGCGAGCCTCGTAGCGGATGCCGCTT‑CCCGTGGTCACATCTCGTGCCTGACTACTAGTGGACGTAACGGTGGCGCTTTGGAAATCACTGCTTCCGGTACTCGCTTTCTGAAACGAATGGGAGGATG‑TGTCTAATGTCTCGTGCCCTTGTGACTATTCCACGCGATGTGTGGAACGatat                                       >  1:821437/2‑301 (MQ=255)`  
                                                                                                                                                                                                                                                                      `aCTCTTTCAAACAATCTCCTGCAACAGTACGGGAGGTGTTCTGATGTCTGACTACCTGAAAGTGCTGCAAGCAATCAAAAGTTGCCCTAAGACTTTCCAGTCCAACTATGTACGGAACAATGCTAGCCTCGTAGCGGAGGCCGCTT‑CCCGTGGTCACATCTCGTGCCTGACTACTAGTGGACGTAACGGTGGCGCTTGGGAAATCACTTCTTCCGGTACTCGCTTTCTGAAACGAATTGGAGGATG‑TGTCTAATGTCTCGTGACCTTGTGACTATTCCACGCGATGTGTGGAACGATATAc                                     <  1:655952/301‑1 (MQ=255)`  
                                                                                                                                                                                                                                                                      `aCTATTTCAAACAAACTCCTGCAACAGTACGGGAGGTGTTCTGATGTCTGACTACCTGAAAGTGCTGCAAGCAATCAAAAGTTGCCTTAAGACTGTCCAGTCCAACTATGTACGGAACAATGCGAGCCTCGTAGCGGAGGCCGCTT‑CCCGTGGTCACATCTCGTGCCTGACTACTAGTTGACGTAACGGTGGCGCTTGGGAAATCACTGCTTCCGGTACTCGCTTTCTGAAACGAATGGGAGGATG‑TGTCTAATGTCTCGTGACCTTGTGACTATTCCACGCAATGTGTGGAACGATATAc                                     <  1:157525/301‑1 (MQ=255)`  
                                                                                                                                                                                                                                                                        `tctTTCAAACAATCTCCTGTAACAGTACGGGAGGTGTTCTGATGTCTGACTACCTGAAAGTGCTGCAAGCAATCAAAAGTTGCCCTAAGACTTTCCAGTCCAACTATGTACGGAACAATGCGAGCCTCGTAGCGGAGGCCGCTT‑CCCGTGGTCACATCTCGTGCCTGACTACTAGTGGACGTAACGGTGGCGCTTGGGAATTCACTGCTTCCGGTACTCGCTTTCTGAAACGAATGGGAGGATG‑TGTCTAATGTCTCGTGACCTTGTGACTATTCCACGCGATGTGTGGAACGATATACAg                                   <  1:356506/301‑1 (MQ=255)`  
                                                                                                                                                                                                                                                                         `ctTTCAAACAATCTCCTGCAACAGTACGGGAGGTGTTCTGATGTCTGACTACCTGAAAGTGCTGCAAGCAATCAAAAGTTGCCATAAGACTTTCCAGTCCAACTATGTACGGAACAATGCGAGCCTCGTAGCGGAGGCCGCTT‑CCCGTGGTCACATCTCGTGCCTGACTACTAGTGGACGTAACGGTGGCACTTGGGAAATCACTGCTTCAGGTACTCGCTTTCTGAAACGAATGGGAGGATG‑TGTCTAATGTCTCGTGACCTTGTGACTATTCCACGCGactgtctcttatacacatctc                                  >  1:469727/1‑281 (MQ=255)`  
                                                                                                                                                                                                                                                                         `ctTTCAAACAATAGCCTGCAACAGTACGTGAGGTGTTCTGATGTCTGACTACCGGAAAGTGCTGCAAGCAATCAAAAGTTGCCCGAAGACTTTCCAGTCCAACTATGTACGGAACAATGCGAGCCTCGTAGCGGAGGCCGCTT‑CCCGTGGTCACATCTCGTGCCTGACTACTAGTGGACGTAACGGTGGCGCTTGGGAAATCACTGCTTCCGGTACTCGCTTTCTGAAACGAATGGGAGGATG‑TGTCTAATGTCTCGTGACCTTGTGACTATTCCACGCGATGTGTGGAACGATATACAgg                                  <  1:345959/301‑1 (MQ=255)`  
                                                                                                                                                                                                                                                                           `ttCAAACAATCTCCTGCAACAGTACGGGAGGTGTTCTGATGTCTGACTACCTGAAAGTGCTGCAAGCAATCAAAATTTGCCCTAATACTTTCCAGTCCAACTATGTACGGAACAATGCGAGCCTCGTAGCGGATGCCGCTT‑CCCGTGGTCACATCTCGTGCCTGACTACTAGTTGACGTAACGGTGGCGCTTGTGAAATCACTGCTTCCGGTACTCGCTTTCTGAAACGAATGGGAGGATG‑TGTCTAATGTCTCGTGACCTTGTGACTATTCCACGCGATGTGTGGAACGATATACAGGGc                                <  1:522595/301‑1 (MQ=255)`  
                                                                                                                                                                                                                                                                                `ctcggagatgtgtataagagacagggTGTTCTGATGTCTGACTACCTGAAAGTGCTGCAAGCAATCAAAAGTTGCCCTAAGACTTTCCAGTCCAACTATGTACGGAACAATGCGAGCCTCGTAGCGGAGGCCGCTT‑CCCGTGGTCACATCTCGTGCCTGACTACTAGTGGACGTAACGGTGGCGCTTGGGAAATCACTGCTTCCGGTACTCGCTTTCTGAAACGAATGGGAGGATG‑TGTCTAATGTCTCGTGACCTTGTGACTATTCCACGCGATGTGTGGAACGATATACAGGGCTACAt                           <  1:404843/277‑1 (MQ=255)`  
                                                                                                                                                                                                                                                                                `accaTCTCCTGCAACAGTACGGGAGGTGTTCTGATGTCTGACTACCTGAAAGTGCTGCAAGCAATCAAAAGTTGTCCTAAGACTTTCCAGTCCAACTATGTACGGAACAATGCGAGCCTCGTAGCGGAGGCCGCTT‑CCCGTGGTCACATCTCGTGCCTGACTACTAGTGGACGTAACGGTGGCGCTTTGGAAATCACTGCTTCCGGTACTCGCTTTCTGAAACGAATGGGAGGATG‑TGTCTAATGTCTCGTGACCTTGTGACTATTCCATGCGATGTGTGGAACGATATACAGGGCTACAt                           <  1:771294/298‑1 (MQ=255)`  
                                                                                                                                                                                                                                                                                    `tctcCTGCAACAGTACGGGAGGTTTTCTGATGTCTGACTACCTGAAAGTGCTGCAAGCAATCAAAAGTTGCCCTAAGACTTTCCAGTACAACTATGTACGGAACAATGCGAGCCTCGTAGCGGAGGCCGCTT‑CACGTGGTCACATCTCGTGCCTGACTACTAGTGGACGTAACGGTGGCGCTTGGGAAATCACTGCTTCCGGTACTCGCTTTCTGAAACGAATGGGAGGATG‑TGTCTAATGTCTCGTGACCTTGTGACTATTCCACGCGATGTGTGGAACGATATACAGGGCTACATCGAc                       <  1:129283/301‑1 (MQ=255)`  
                                                                                                                                                                                                                                                                                      `tcCTGCAACAGTACGGGAGGTGTTCTGATGTCTGACTACCTGAAAGTGCTGCAAGCAATCAAAAGTTGCCCTAAGACTTTCCAGTCCAACTATGTACGGAACAATGCGAGCCTCGTAGCGGAGGCCGCTT‑CCCGTGGTCACATCTCGTGCCTGACTACTAGTGGACGTAACGGTGGCGCTTGGGAAATCACTGCTTCCGGTACTCGCTTTCTGAAACGAATGGGAGGATG‑TGTCTAATGTATCGTGACCTTGTGACTATTCCACGCGCtgtctcttatacacatctccgagcccacgagac                     >  1:207348/1‑271 (MQ=255)`  
                                                                                                                                                                                                                                                                                      `tcCTGCAACAGTACGGGAGGTGTTCTGATGTCTGACTACCTGAAAGTGCTGCAAGCAATCAAAAGTTGCCCTAAGACTTTACAGTCCAACTATGTACGGAACAATGCGAGCCTCGTAGCGGAGGCCGCTT‑CCAGTGGTCACATCTCGTGCCTGACTACTAGTGGACGTAACGGTGGCGCTTGGGAAATCACTGCTTCCGGTACTCGCTTTCTGAAACGAATGGGAGGATG‑TGTCTAATGTCTCGTGACCTTGTGACTATTCCACGCGATGTGTGGAACGATATACAGGGCTACATCGActc                     <  1:646716/301‑1 (MQ=255)`  
                                                                                                                                                                                                                                                                                       `ccTGCAACAGTACGGGAGGTGTTCTGATGTCTGACTACCTGAAAGTGCTGCAAGCAATCAAAAGTTGCCCTAAGACTTTACAGTCCAACTATGTACGGAACAATGTGAGCCTCGTAGCGGAGGCCGCTT‑CCCGTGGTCACATCTCGTGCCTGACTACTAGTGGACGTAACGGTGGCGCTTGGGAAATCACTGCTTCCGGTACTCGCTTTCTGAAACGAATGGGAGGATG‑TGTCTAATGTCTCGTGACCTTGTGACTATTCCACGCGATGTGTGGAACGATATACAggctgtctcttataca                    >  1:177781/1‑287 (MQ=255)`  
                                                                                                                                                                                                                                                                                           `cAACAGTACGGGAGGTGTTCTGATGTCTGACTACCTGAAAGTGCTGCAAGCAATCAAAAGTTGCCATAAGACTTTCCAGTCCAACTATGTACGGAACAATGCGAGCCTCGTAGCGGAGGCCGCTT‑CCCGTGGTCACATCTCGTGCCTGACTACTAGTGGACGTAACGGTGGCGCTTGGGAAATCACTGCTTCAGGTACTCGCTTTCTGAAACGAATGGGAGGATG‑TGTCTAATGTCTCGTGACCTTGTGACTATTCCACGCGATGTGTGGAACGATATACAGGGCTACATCGACTCTCTgg                <  1:705529/301‑1 (MQ=255)`  
                                                                                                                                                                                                                                                                                           `aaaCAGTACGGGAGGTGTTCTGATGTCTGACTACCTGAAAGTGCTGCAAGCAATCAAAAGTTGCCCTAAGACTTTCCAGTACAACTATGTACGGAACAATGCGAGCCTCGTAGCGGAGGCCGCTT‑CCCGTGGTCACATCTCGTGCCTGACTACTAGTGGACGTAACGGTGGCGCTTGGGAAATCACTGCTTCCGGTACTCGCTTTCTGAAACGAATGGGAGGATG‑TGTCTAATGTCTCGTGACCTTGTGACTATTCCACGCGATGTGTGGAACGATATACAGGGCTACATCGACTCTCTgg                <  1:9034/300‑1 (MQ=255)`  
                                                                                                                                                                                                                                                                                              `aaGTACGGGAGGTGTTCTGATGTCTGACTACCTGAAAGTGCTGCAAGCAATAAAAAGTTGCCCTAAGACTTTCCAGTCCAACTATGTACGGAACAATGCGAGCCTCGTAGCGGAGGCCGCTT‑CCCGTGGTCACATTTCGTGCCTGACTACTAGTGGACGTAACGGTGGCGCTTGGGAAATCACTGCTTCCGGTACTCGCTTTCTGAAACGAATGGGAGGATG‑TGTCTAATGTCTCGTGACCTTGTGACTATTCCACGCGATGTGTGGAACGATATACAGGGCTACATCGACTCTCTAGAAc             <  1:77901/300‑1 (MQ=255)`  
                                                                                                                                                                                                                                                                                                `gTACGGGAGGTGTTCTGATGTCTGACTACCTGAAAGTGCTGCAAGCAATCAAAAGTTGCCCTAAGACTTTCCAGTCCAACTATGTACGGAACAATGCGAGCCTCGTAGCTGAGGCCGCTT‑CCCGTGGTCACATCTCGTGCCTGACTACTAGTGGACGTAACGGTGGCGCTTGGGAAATCACTGCTTCCGGTACTCGCTTTCTGAAACGAATTGGAGGATG‑TGTCTAATGTCTCGTGACCTTGTGACTATTCCACGCGATGTGTGGAACGATATACAggctgtctcttatacacatctccga           >  1:572154/1‑278 (MQ=255)`  
                                                                                                                                                                                                                                                                                                `gTACGGGAGGTGTTCTGATGTCTGACTACCTGAAAGTGCTGCAAGCAATCAAAAGTTGCCCTAAGACTTTCCAGTCCAACTATGTACGGAACAATGCGAGCCTCGTAGCGGAGGCCGCTT‑CCCGTGGTCACATCTCGTGCCTGACTACTAGTGGACGTAACGGTGGCGCTTGGGAAATCACTGCTTCCGGTACTCGCTTTCTGAAACGAATGGGAGGATG‑TGTCTAATGTCTCGTGACCTTGTGACTATTCCACGCGATGTGTGGAACGATATACAGGGCTACATCGACTCTCTGGAACgg           >  1:629344/1‑300 (MQ=255)`  
                                                                                                                                                                                                                                                                                                `atACAGGAGGTGTTCTGATGTCTGACTACCTGAAAGTGCTGCAAGCAATCAAAAGTTGCCCTAAGACTTTCCAGTCCAACTATGTACGGAACAATGCGAGCCTCGTAGCGGAGGCCGCTT‑CCCGTGGTCACATCTCGTGCCTGACTACTAGTTGACGTAACGGTGGCGCTTGGGTAATCACTGCTTCCGGTACTCGCTTTCTGAAACGAATGGGAGGATG‑TGTCTAATGTCTCGTGACCTTGTGACTATTCCACGCGATGTGTGGAACGATATACAGGGCTACATctgtctcttatacaca           >  1:300553/2‑286 (MQ=255)`  
                                                                                                                                                                                                                                                                                                  `aCGGGAGGTGTTCTGATGTCTGACTACCTGAAAGTGCTGCAAGCAATCAAAGGTTGCCCTAAGACTTTCCAGTCCAACTATGTACGGAACAATGCGAGCCTCGTAGCTGAGGCCGCTT‑CCCGTGGTCACATCTCGTGCCTGACTACTAGTGGACGTAACGGTGGCGCTTGGGAAATCACTGCTTCCGGTACTCGCTTTCTGAAACGAATGGGAGGATG‑TGTCTAATGTCTCGTGACCTTGTGACTATTCCACGCGATGTGTGGAACGATATACAGGGCTACATCGACTCTCTAGAACCTgt         >  1:83701/1‑300 (MQ=255)`  
                                                                                                                                                                                                                                                                                                    `gggAGGTGTTCTGATGTCTGACTACCTGAAAGTGCTGCAAGCAATCAAAAGTTGCCATAAGACTTTCCAGTCCAACTATGTACGGAACAATGCGAGCCTCATAGCGGAGGCCGCTT‑CCCGTGGTCACATCTCGTGCCTGACTACTAGTGGACGTAACGGTGGCGCTTGGGAAATCACTGCTTCCGGTACTCGCTTTCTGAAACGAATGGGAGGATG‑TGTCTAATGTCTCGTGGCCTTGTGACTATTCCACGCGATGTGTGGAACGATATACAGGGCTACATctgtctcttatacacatctc       >  1:263767/1‑282 (MQ=255)`  
                                                                                                                                                                                                                                                                                                     `ggAGGTGTTCTGATGTCTGACTACCTGAAAGTGCTGCAAGCAATCAAAAGTTGCCATAAGACTTTCCAGTCCAACTATGTACGGAACAATGCGAGCCTCGTAGCGGAGGCCGCTT‑CCCGTGGTCACATCTCGTGCCTGACTACTAGTGGACGTAACGGTGGCGCTTGGGAAATCACTGCTTCCGGTACTCGCTTTCTGAAACGAATGGGAGGATG‑TGTCTAATGTCTAGTGACCTTGTGACTATTCCACGCGATGTGTGGAACGATATACAGGGCTACATCGACTCTCTGGAACCTgtctc      >  1:748267/1‑297 (MQ=255)`  
                                                                                                                                                                                                                                                                                                      `gAGGTGTTCTGATGTCTGACTACCTGAAAGTGATGCAAGCAATCAAAAGTTGCCCTAAGACTTTCCAGTCCAACTATGTACGGAACAATGCGAGCCTCGTAGCGGAGGACGCTT‑CCCGTGGTCACATCTCGTGCCTGACTACTAGTGGACGTAACGGTGGCGCTTGGGAAATCACTGCTTCCGGTACTCGCTTTCTGAAACGAATGGGAGGATG‑TGTCTAATGTCTCGTGACCTTGTGACTATTCCACGCGATGTGTGGAACGATATACAGGGCtctgtctcttatacacatctccgagcc     >  1:437423/1‑275 (MQ=255)`  

                                                                                                                                                                                                                                                                                                          `|`                                                                                                                                                                                                                                                                                                               
`ATTATTATGGCTATGACAAAGAAATTTAAAGTGTCCTTCGACGTTACCGCAAAGATGTCGTCTGACGTTCAGGCAATCTTAGAGAAAGATATGCTGCATCTATGTAAGCAGGTCGGCTCAGGTGCGATTGTCCCCAATGGTAAACAGAAGGAAATGATTGTCCAGTTCCTGACACACGGTATGGAAGGATTGATGACATTCGTAGTACGTACATCATTTCGTGAGGCCATTAAGGACATGCACGAAGAGTATGCAGATAAGGACTCTTTCAAACAATCTCCTGCAACAGTACGGGAGGTGTTCTGATGTCTGACTACCTGAAAGTGCTGCAAGCAATCAAAAGTTGCCCTAAGACTTTCCAGTCCAACTATGTACGGAACAATGCGAGCCTCGTAGCGGAGGCCGCTT‑CCCGTGGTCACATCTCGTGCCTGACTACTAGTGGACGTAACGGTGGCGCTTGGGAAATCACTGCTTCCGGTACTCGCTTTCTGAAACGAATGGGAGGATG‑TGTCTAATGTCTCGTGACCTTGTGACTATTCCACGCGATGTGTGGAACGATATACAGGGCTACATCGACTCTCTGGAACGTGAGAACGAT  >  NC_001604.1/16845‑17442`

  

Alignment Legend

Aligned base mismatch/match (shaded by quality score): `ATCG/ATCG` `< 3 ≤` `ATCG/ATCG` `< 19 ≤` `ATCG/ATCG` `< 29 ≤` `ATCG/ATCG` `< 37 ≤` `ATCG/ATCG` `< 38 ≤` `ATCG/ATCG`

Unaligned base: `atcg`    Masked matching base: `atcg`    Alignment gap: `‑`    Deleted base: `‑`

Reads not counted as support for junction

read\_name Not counted due to insufficient overlap past the breakpoint.

read\_name Not counted due to not crossing MOB target site duplication.
